# Editorial: Autophagy in the central nervous system: Focus on neurons, glia and neuron-glia interactions

**DOI:** 10.3389/fcell.2022.1036587

**Published:** 2022-10-12

**Authors:** Maria Jimenez-Sanchez, Olatz Pampliega, Sandra-Fausia Soukup

**Affiliations:** ^1^ Maurice Wohl Clinical Neuroscience Institute, King’s College London, London, United Kingdom; ^2^ Departamento de Neurociencias, Achucarro Basque Center for Neurosciences, Universidad del País Vasco (UPV/EHU), Leioa, Spain; ^3^ University Bordeaux, CNRS, IMN, UMR 5293, Bordeaux, France

**Keywords:** autophagy, neurodegeneration, neurons, glia, central nervous system

Autophagy is a fundamental catabolic recycling process of the cell and plays an essential role in brain physiology and pathology. We can differentiate three major autophagy types that all degrade there cargo *via* the endo-lysosomal system; chaperone-mediated autophagy (CMA), microautophagy, and macroautophagy. CMA and microautophagy use cytosolic chaperone proteins to transport proteins directly to lysosomes or endosomes respectively. CMA requires the LAMP2A receptor on lysosomes for substrate binding, while microautophagy transfers the cargo by invagination of lysosomal and endosomal membranes. In contrast, macroautophagy (here after called autophagy) consists in the formation of an autophagic membrane that engulfs cytoplasmic material that than fuses with the lysosome to degrade the content.

In this Research Topic, we bring together, a collection of articles that highlight the role of autophagy in the brain with particular view on neurons, neuroglia and synaptic compartments, dysregulation of autophagy in neurodegeneration, methods to detect, analyze and quantify autophagy as well as points of therapeutic opportunities in neurodegenerative disease.

In neurons, autophagy presents cell specific adaptations. Neurons are highly polarized post-mitotic cells that are particularly sensitive to oxidative stress and the accumulation of dysfunctional and toxic proteins. Moreover, presynaptic compartments can lay sometimes fare away from the soma, have limited local translation mechanisms and these compartments host the areas where synaptic vesicles fuse with the plasma membrane to release neurotransmitter for communication with post synaptic site. The work from Decet and Verstreken summaries our current understanding of autophagosomal biogenesis at presynaptic terminals and how autophagy is connected to neurotransmission (Decet and Verstreken). Autophagosomal biogenesis is induced at presynaptic terminals by metabolic signals (e.g., Amino acid deprivation) and prolonged neuronal activity and it is locally regulated by presynaptic enriched proteins (Soukup et al., 2016; Okerlundk et al., 2017; Vanhauwaert et al., 2017; Bademosi et al., 2022; Hernandez-Diaz et al., 2022). The authors discuss the complex relationship between autophagy and neurotransmission, and review how autophagy at presynaptic terminals modulates neurotransmission *via* synaptic vesicle turnover/recycling and intracellular calcium buffering *via* tubular ER. Increasing of our knowledge about synaptic autophagy would not be only critical to better understand the modulation of neurotransmission particularly in neuronal circuits but also for our understanding of neurodegeneration.

To address this knowledge gap, in this Research Topic Sanchez-Mirasierra et al. establish a novel method to quantify autophagy levels in particular cell types in the brain or in cellular compartments such as the presynaptic-terminals. The authors developed an ImageJ base image analysis “Autophagoquant” and “Exoquant” that completely automatizes the quantification of autophagosomes and exosomes at presynaptic terminals in *Drosophila melanogaster,* through the analysis of fluorescent tagged autophagy marker LC3/Atg8 by fluorescent microscopy and without the researchers decision-making intervention (Sanchez-Mirasierra et al.).

Autophagic alterations are common in many neurodegenerative diseases such as Parkinson’s disease (PD), the second most common neurodegenerative disease after Alzheimer’s disease. PD is characterized by neurological and motor dysfunction that can go along with non-motor symptoms. Currently there is no cure for PD and therapeutic approaches only ameliorate motor symptoms. The work of Sanchez-Mirasierra et al. recapitulates the function of PD-causative proteins in the macroautophagy pathway (Sanchez-Mirasierra et al.). This work further describe potential drugable targets and current clinical trials targeting the macroautophagy pathway to treat PD. The authors explain that macroautopaghy offers various therapeutic opportunities for treating PD, even for non-motor symptoms, but highlight the need to increase our current knowledge about autophagy and the function of PD causative proteins in other pathways to evaluate side effects when targeting these proteins.

Primary identified as a process to degrade proteins and organelles, autophagy is now also recognized to participate also in RNA homeostasis. Houghton et al. summaries the role of autophagy in RNA homeostasis, especially in the context of amyotrophic lateral sclerosis (ALS) and frontotemporal dementia (FTD) (Houghton et al.). ALS and FTD are neurodegenerative diseases caused by a repeat expansion of the C9orf72 gene and both diseases display alterations of RNA catabolisms. The authors discuss how in genetic forms of ALS and FTD, autophagic alterations lead to accumulation of pathological RNA granules and consequently protein aggregation in the cytoplasm.

The work of Gomez et al. summaries the role of autophagy in the clearance of advanced glycation end products (AGEs) to avoid glycative stress that is increasingly recognized during aging and neurodegeneration (Gómez et al.). AGEs are modified forms of lipids or proteins that become oxidized and glycated upon exposition to sugars. Neurons and glia have a high exchange of metabolites and glia cells show a higher accumulation of AGEs. Indeed, autophagy in glia cells is fundamental to maintain glial homeostasis but also for recycling metabolites that are transported to neurons. The authors explain how under physiological conditions, glia show higher detoxification capabilities and counteract glycation by expressing higher levels of the deglycase enzyme DJ-1, a PD causative protein. In contrast, higher levels of AEG are found in PD patients and therefore the degradation of AEGs *via* autophagy is discussed as a therapeutic strategy.

Adequate levels of autophagy are essential for brain physiopathology. However, the precise mechanisms linking defects in autophagy and neurodegeneration are still not well characterized. Moreover, regulation of autophagy can be cell- and compartment-specific, and autophagy dysfunction may contribute differently to disease pathogenesis and at different disease stages depending on the brain cell type. While the field has expanded enormously since autophagy was first described in the early 90s (Ohsumi, 2014), recognizing autophagy function and regulation in each brain cell type requires further attention. To understand cell-specific regulation of autophagy in glia types and to decipher how autophagy participates in the functional coupling between neuron and glia will be essential to uncover the role of autophagy in neurodegenerative and other brain diseases but also in physiological processes such as memory and plasticity.

## References

[B1] Bademosi A. T. D. Decet M. Kuenen S. Calatayud C. Swerts J. Gallego S. F. (2022). EndophilinA-dependent coupling between activity-dependent calcium influx and synaptic autophagy is disrupted by a Parkinson-risk mutation. bioRxiv Prepr, 1. 10.1101/2022.04.29.490010 PMC10166451 36827984

[B2] Hernandez-Diaz S. Ghimire S. Sanchez-Mirasierra I. Montecinos-Oliva C. Swerts J. Kuenen S. (2022). Endophilin-B regulates autophagy during synapse development and neurodegeneration. Neurobiol. Dis. 163, 105595. 10.1016/j.nbd.2021.105595 34933093

[B3] Ohsumi Y. (2014). Historical landmarks of autophagy research. Cell Res. 24, 9–23. 10.1038/cr.2013.169 24366340 PMC3879711

[B4] Okerlundk N. D. Schneider K. Leal-Ortiz S. Montenegro-Venegas C. Kim S. A. Garner L. C. (2017). Bassoon controls presynaptic autophagy through Atg5. Neuron 93, 897–913. e7. 10.1016/j.neuron.2017.01.026 28231469

[B5] Soukup S.-F. F. Kuenen S. Vanhauwaert R. Manetsberger J. Hernández-Díaz S. Swerts J. (2016). A LRRK2-dependent EndophilinA phosphoswitch is critical for macroautophagy at presynaptic terminals. Neuron 92, 829–844. 10.1016/j.neuron.2016.09.037 27720484

[B6] Vanhauwaert R. Kuenen S. Masius R. Bademosi A. Manetsberger J. Schoovaerts N. (2017). The SAC1 domain in synaptojanin is required for autophagosome maturation at presynaptic terminals. EMBO J. 36, 1392–1411. 10.15252/embj.201695773 28331029 PMC5430236

